# Role of the I16-D194 ionic interaction in the trypsin fold

**DOI:** 10.1038/s41598-019-54564-6

**Published:** 2019-12-02

**Authors:** Bosko M. Stojanovski, Zhiwei Chen, Sarah K. Koester, Leslie A. Pelc, Enrico Di Cera

**Affiliations:** Edward A. Doisy Department of Biochemistry and Molecular Biology, Saint Louis University School of Medicine, St. Louis, MO 63104 USA

**Keywords:** Structural biology, Enzyme mechanisms

## Abstract

Activity in trypsin-like proteases is the result of proteolytic cleavage at R15 followed by an ionic interaction that ensues between the new N terminus of I16 and the side chain of the highly conserved D194. This mechanism of activation, first proposed by Huber and Bode, organizes the oxyanion hole and primary specificity pocket for substrate binding and catalysis. Using the clotting protease thrombin as a relevant model, we unravel contributions of the I16-D194 ionic interaction to Na^+^ binding, stability of the transition state and the allosteric E*-E equilibrium of the trypsin fold. The I16T mutation abolishes the I16-D194 interaction and compromises the architecture of the oxyanion hole. The D194A mutation also abrogates the I16-D194 interaction but, surprisingly, has no effect on the architecture of the oxyanion hole that remains intact through a new H-bond established between G43 and G193. In both mutants, loss of the I16-D194 ionic interaction compromises Na^+^ binding, reduces stability of the transition state, collapses the 215–217 segment into the primary specific pocket and abrogates the allosteric E*-E equilibrium in favor of a rigid conformation that binds ligand at the active site according to a simple lock-and-key mechanism. These findings refine the structural role of the I16-D194 ionic interaction in the Huber-Bode mechanism of activation and reveal a functional linkage with the allosteric properties of the trypsin fold like Na^+^ binding and the E*-E equilibrium.

## Introduction

Members of the trypsin family of proteases participate in key physiological processes ranging from blood coagulation to apoptosis^1^. Catalytic activity is brokered by three highly conserved residues that correspond to H57, D102 and S195 in the chymotrypsinogen numbering^2^. Additional residues contribute to substrate recognition within the active site region^3^. The acidic D189 engages the basic Arg/Lys residue at the P1 position of substrate^4^. Two backbone N atoms from the catalytic S195 and neighbor G193 define the so called oxyanion hole and help stabilize the tetrahedral intermediate during the catalytic cycle. West to the entrance of the active site region, the entire 215–217 segment defines recognition subsites for the P2 and P3 positions of substrate. This segment is of interest insofar as it may assume alternative conformations, closed (E*) and open (E), that control access to the active site and the primary specificity pocket^5^. The Protein Data Bank (PDB) provides strong support for the E*-E equilibrium^5,6^, and so do rapid kinetics studies of ligand binding^7,8^.

A common theme in the trypsin family is that proteases are synthesized as inactive zymogens and then converted to the active enzyme by cleavage at residue R15^9^ that generates a new N-terminus, typically at residue I16. A new H-bonds then forms with the side chain of residue D194 leading to organization of the primary specificity pocket, the oxyanion hole and other critical epitopes of the active site to enable substrate binding and catalysis^1,3,10^. This is the celebrated Huber-Bode mechanism of zymogen activation^9^ where the critical H-bond between I16 and D194 provides the driving force for organizing the entire architecture of the active protease. The mechanism is centerpiece in our current understanding of protease biology and particularly of enzyme cascades^11^. The role of the I16-D194 interaction is so crucial that all alternative modes of zymogen activation that bypass cleavage at R15 target D194 to organize the active site. Single chain tissue-type plasminogen activator features catalytic activity independent of proteolytic cleavage at residue 15 through a H-bond established by K156 with D194^12^. A similar interaction is observed for the plasminogen activator in the saliva of *D. rotundus*^13^. Bacteria have evolved proteins like streptokinase^14^ and staphylocoagulase^15^ that engage D194 directly in their target zymogens plasminogen or prothrombin, thus bypassing the canonical activation of the host fibrinolytic and coagulation cascades. Protein engineering has endorsed a similar strategy by hijacking D194 with zymogen activator peptides developed by phage display^16^. More recently, a linkage has been suggested between the Huber-Bode mechanism and the pre-existing E*-E equilibrium of the trypsin fold. Specifically, the zymogen to protease transition has been shown to gradually convert the closed conformation E* to the open form E, leading to greatly enhanced substrate binding and catalysis^8^. Hence, the I16-D194 ionic interaction contributes to multiple features of the trypsin fold and deserves attention.

Much information on the role of residue D194 in the mature protease and its linkage with structural determinants of binding, catalysis and conformational equilibria is expected from studies of the obvious substitution D194A, that should abrogate the critical H-bond with I16 in the Huber-Bode mechanism and force the fold to assume a zymogen-like conformation. Surprisingly, this simple expectation has not been tested experimentally and there are no structures of the D194A mutant for any trypsin-like protease deposited in the PDB. The role of D194 has so far been explored through conservative substitutions such as D194E or D194N combined with mutations/deletions of I16, or with structures bound to inhibitors such as BPTI^17–19^. None of these structures provides information on the architecture of the free form of the protease with the I16-D194 ionic interaction perturbed. Other studies have provided valuable information on the functional consequences of replacing I16, but have not documented the structural basis of the observed effects^20–22^. Here, we investigate the role of the I16-D194 H-bond using both structural and functional studies of the clotting protease thrombin, one of the best characterized members of the trypsin family of proteases^23^. The results reveal new features controlled by the I16-D194 ionic interaction.

## Results

### Crystal structure of the D194A mutant

**Figure 1:**
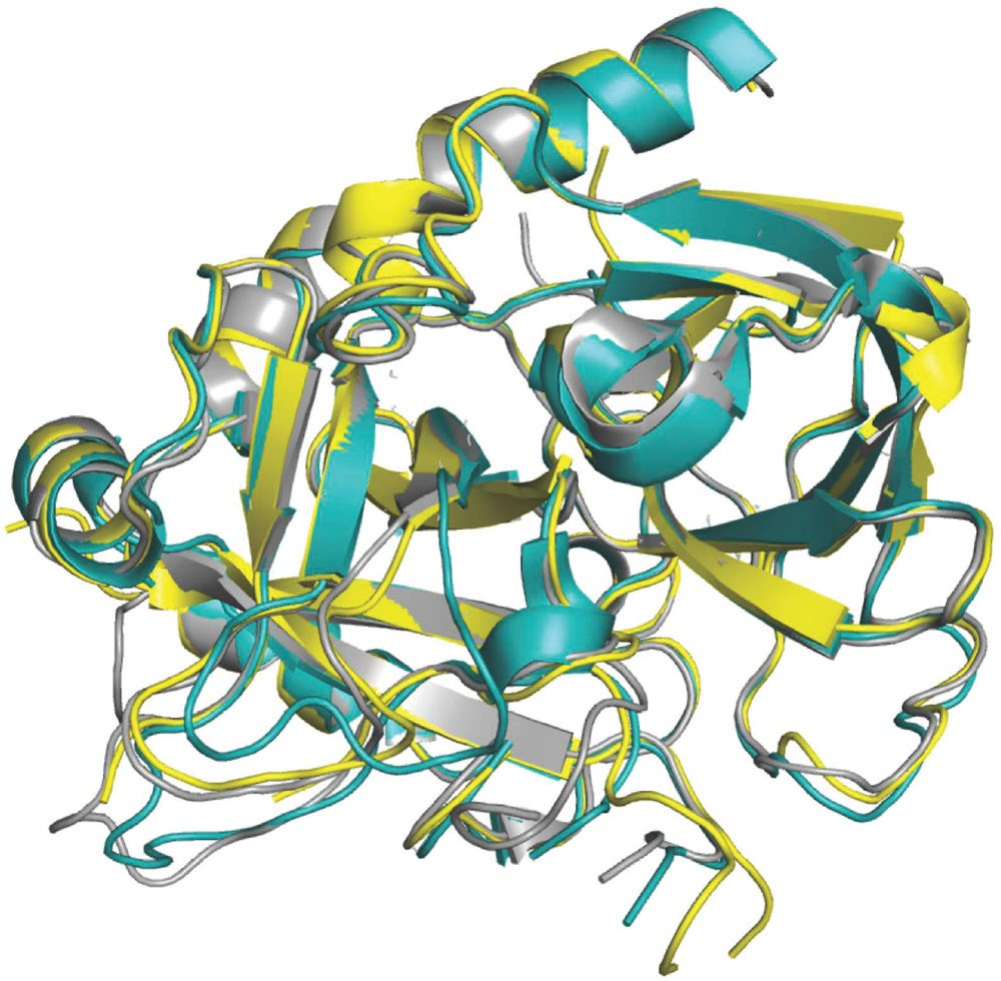
Crystal structure of the D194A mutant (yellow) aligned with the structures of thrombin in the E (rmsd = 0.49 Å; PDB entry 1SGI, grey) and E* (rmsd = 0.45 Å; PDB entry 2GP9, cyan) conformations.

**Figure 2:**
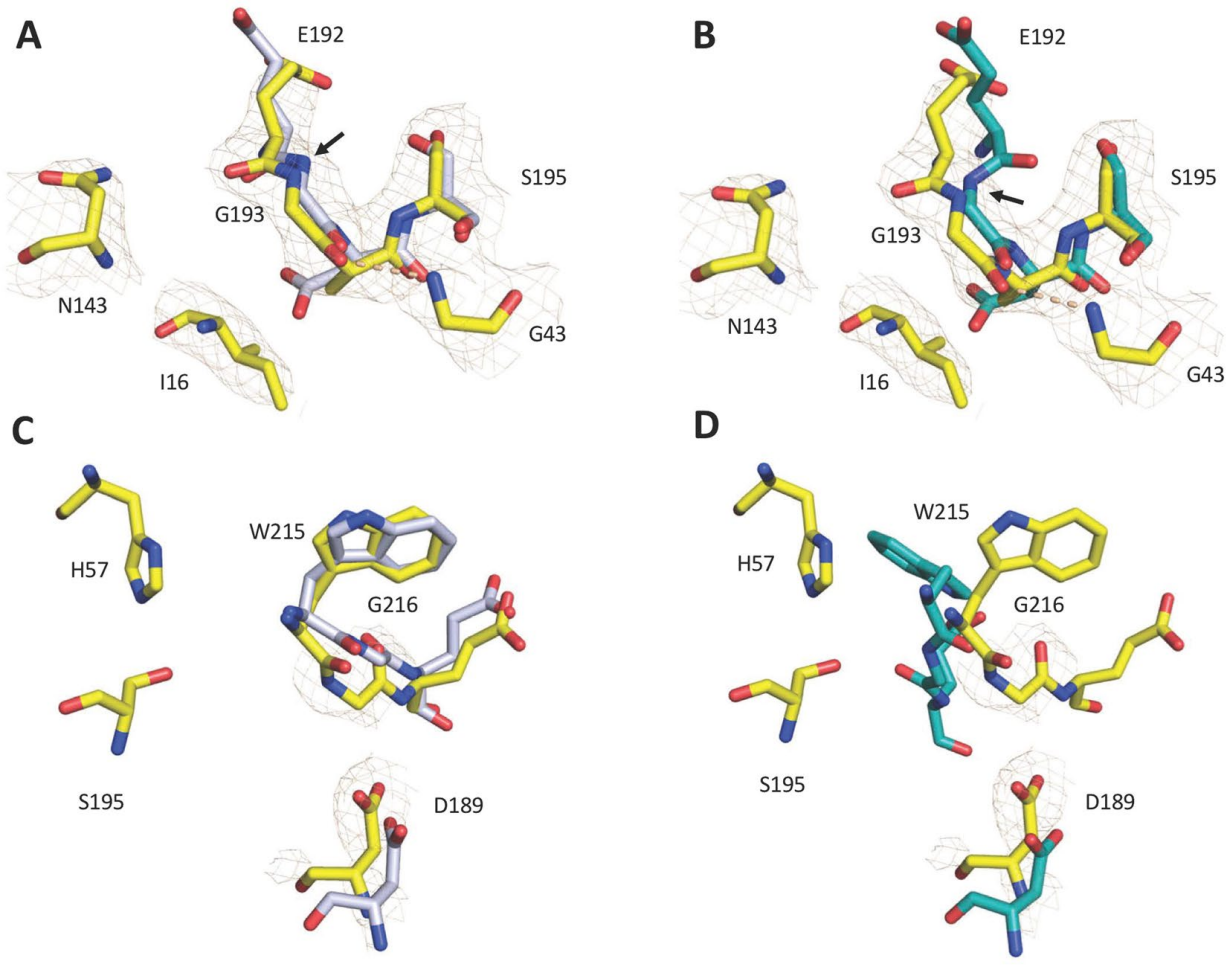
(**A–D**) Alignment of the 192–195 segment of the D194A mutant (yellow) with the structures of thrombin in the E (**A**) and E* (**B**) conformations. Despite abrogation of the N143-E192 H-bond, the integrity of the oxyanion hole is preserved and the 192–193 peptide bond (designated with an arrow) assumes the conformation seen in the E form (A) instead of the flipped conformation seen in the E* form (**B**). A novel H-bond between G43 and G193 (dashed lines) contributes to the structural integrity of the oxyanion hole. Alignment of the residues that define the primary specificity pocket in the D194A mutant (yellow) with those of the E (**C**) and E* (**D**) forms of thrombin shows a shift in the positions of D189 and G216, which assumes a conformation intermediate between that of the E and E* forms. In all panels, PDB accession codes 1SGI (grey) and 2GP9 (cyan) were used for the E and E* conformations of thrombin.

The crystal structure of the thrombin mutant D194A was solved in the free form at a resolution of 2.8 Å and final *R*_free_ = 0.29 (Table 1). Although resolution is not as high as for the I16T mutant (see below), it gives confidence to the interpretation of the main features observed in the structure that is similar to that of wild-type in the E form^24^ (Fig. 1) but with significant disorder in the autolysis loop and parts of the 186-loop around the Na^+^ binding site. Other regions of interest are the segments 141–143, 189–194 and 214–219 that participate in the architecture of the active site region. As expected, the D194A mutation abrogates the critical ionic interaction between the Oδ2 atom of D194 and the amino terminus of I16. The additional H-bond between the Oδ1 atom of D194 and the backbone N atom of G142 is also disrupted, forcing the entire 141–143 segment to undergo a conformational shift of more than 7 Å in the direction of the 70-loop in exosite I. A notable consequence of this shift is the loss of the H-bond between N143 and E192. When this interaction is lost, the 192–193 peptide bond flips and the architecture of the entire 192–194 segment changes into a 3_10_ helix, disrupting the structural integrity of the oxyanion hole. These perturbations have been documented in several systems where the I16-D194 H-bond is intact (i.e., several structures of thrombin^25–30^, the *S. aureus* epidermolytic toxin A^31^, arterivirus nsp4^32^, complement factor B^33^, exfoliative toxin A^34^ and clotting factor VIIa^35^) and thus cannot be assigned to disruption of the I16-D194 ionic interaction. The structure of D194A is peculiar because loss of the N143–E192 H-bond is not accompanied by disruption of the architecture of the oxyanion hole. A compensatory H-bond with G43 stabilizes the conformation of G193 (Fig. 2). The new interaction causes the entire 189–195 segment to shift in the direction of the strand housing G43, and by using the C191–C220 disulfide bond as a hinge, the 215–217 segment is pulled into the active site toward D189 in the primary specificity pocket. The main chain of D189 relinquishes its H-bond interactions with V17 and causes the Oδ2 atom to move nearly 3 Å toward the Cα atom of G216 (Fig. 2). These changes compromise the canonical interactions of residues D189 and G216 with the P1 and P3 residues of substrate^1,3,10^ and presage a significant loss of catalytic activity. Notably, the depth of the primary specificity pocket as measured by the distance between the Oγ atom of the catalytic S195 and the Oδ1 atom of D189 shrinks by 1.6 Å (Fig. 2). The distance between the Nε2 atom of the catalytic H57 and the Oδ2 atom of D189 is similarly reduced. A superimposition of the structure of D194A with the active site inhibitor PPACK in the thrombin-PPACK structure^2,24^ reveals that the backbone of G216 is no longer within H-bonding distance with the P3 residues of the inhibitor.

**Table 1:** Crystallographic data for the thrombin mutants I16T and D194A.

PDB entry	6PXJ	6PXQ
Buffer/salt	200 mM Mg-formate	200 mM Na_2_-phosphate
PEG	3350 (20%)	3350 (20%)
**Data collection:**		
Wavelength (Å)	1.54	1.54
Space group	P2_1_2_1_2	P4_1_2_1_2
Unit cell dimensions (Å)	a = 81.6, b = 151.4, c = 50.6	a = 73.2, b = 73.2, c = 160.7
Molecules/asymmetric unit	2	1
Resolution range (Å)	40–1.7	40–2.8
Observations	426025	132966
Unique observations	68725	10857
Completeness (%)	98.5 (97.3)	94.8 (73.9)
R_sym_ (%)	5.3 (70.3)	14.7 (43.5)
I/σ (I)	23.9 (2.5)	11.7 (1.6)
**Refinement:**		
Resolution (Å)	40–1.7	40–2.8
R_cryst_, R_free_	0.17, 0.20	0.26, 0.29
Reflections (working/test)	65368/3335	10262/559
Protein atoms	4492	2252
Mg^++^ ion	1	—
Solvent molecules	463	0
Rmsd bond lengths^a^ (Å)	0.013	0.008
Rmsd angles^a^ (°)	1.8	1.3
Rmsd ΔB (Å^2^) (mm/ms/ss)^b^	3.03/3.39/4.44	3.71/3.12/2.84
<B> protein (Å^2^)	34.4	90.2
<B> Mg^++^ ion (Å^2^)	37.7	—
<B> solvent (Å^2^)	45.1	—
**Ramachandran plot:**		
Most favored(%)	99.6	99.6
Generously allowed (%)	0.4	0.4
Disallowed (%)	0.0	0.0

^a^Root-mean-squared deviation (Rmsd) from ideal bond lengths and angles and Rmsd in B-factors of bonded atoms. ^b^mm, main chain-main chain; ms, main chain-side chain; ss, side chain-side chain.

### Crystal structure of the I16T mutant

**Figure 3:**
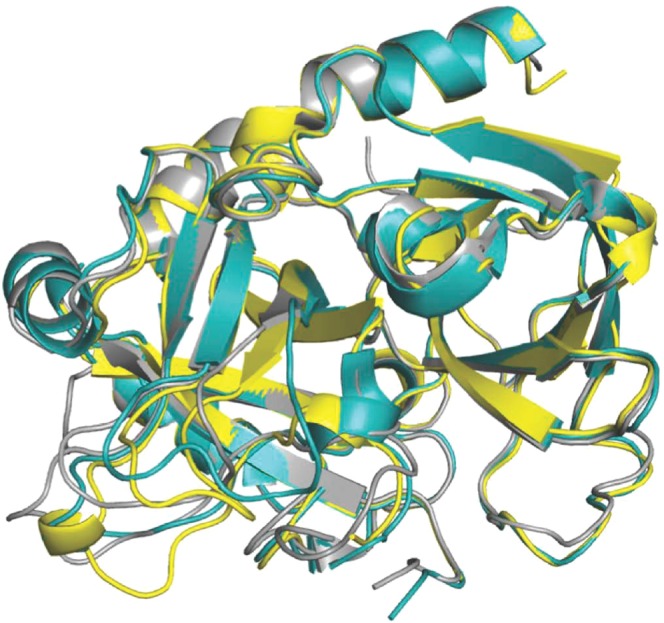
Crystal structure of the I16T mutant (yellow) aligned with the structures of thrombin in the E (rmsd = 0.39 Å; PDB entry 1SGI, grey) and E* (rmsd = 0.37 Å; PDB entry 2GP9, cyan) conformations.

**Figure 4:**
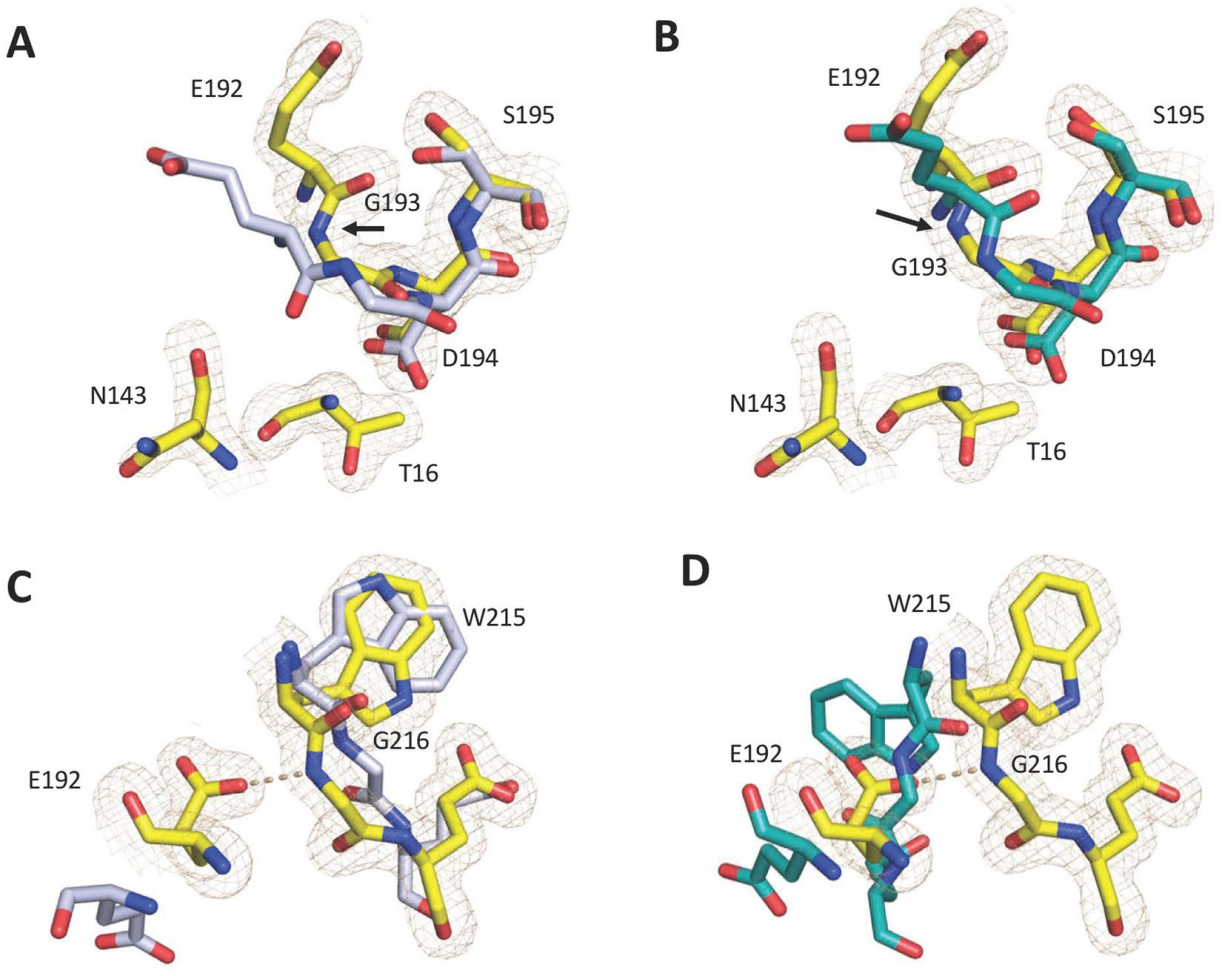
(**A–D**) Alignment of the 192–195 segment of the I16T mutant (yellow) with the structures of thrombin in the E (**A**) and E* (**B**) conformations. As a result of abrogation of the N143-E192 H-bond, the 192–193 peptide bond flips (arrow) and the N atom of G193 points away from that of S195 as observed in the E* form (**B**). Note how the conformation of the 192–193 peptide bond differs from that in E thrombin (**A**). The polar side chain of T16 inserts into the active site within ionic interaction with D194. In contrast to the E form, the active site of the I16T mutant (yellow) is occluded by a partial collapse of the 215–217 segment and a conformational rearrangement of the side chain of E192 (**C**). The restricted accessibility of the active site is comparable to the occlusion observed in the E* conformation (**D**). In all panels, PDB accession codes 1SGI (grey) and 2GP9 (cyan) were used as the E and E* conformations of thrombin.

As an alternative perturbation of the I16-D194 ionic interaction, the I16T mutant was also crystallized in the free form at a resolution of 1.7 Å and final *R*_free_ = 0.20 (Table 1). The structure of I16T is similar to that of wild-type in the E form^24^ but with differences affecting the autolysis loop, the 186- and 220- loops defining the Na^+^ binding site and the 141–143, 189–195 and 214–217 segments around the active site (Fig. 3). The substituted polar side chain of T16 inserts into a hydrophobic cleft in a position similar to that of I16 in the wild-type and its N terminus establishes an ionic interaction with the side chain of D194 (Fig. 4). The position of residue D194 is slightly altered and breaks the H-bond with the backbone N atom of G142, which in turn shifts the 141–143 strand, breaks the N143–E192 H-bond and causes the 192–193 peptide bond to flip (Fig. 4). The resulting perturbation destroys the architecture of the oxyanion hole, unlike what is observed in the structure of the D194A mutant (Fig. 2). Also notable is the position of the side chain of E192, which moves and H-bonds to the backbone N atom of G216. The new interaction pulls the entire 215–217 segment into the active site and seals access to the primary specificity pocket (Fig. 4). These changes are similar to those observed in the E* form of thrombin and several trypsin-like proteases^5,6^. Residue D189 moves and points toward the neighbor autolysis loop and residues 16–19 instead of being directed toward the 215–225 segment as in the wild-type.

### Na^+^ binding

Thrombin is a member of the large family of monovalent cation activated enzymes^36–38^ and specific binding of Na^+^ produces a significant enhancement of catalytic activity toward synthetic^39,40^ and physiologic^41^ substrates. The Na^+^ binding site is nestled between the 186- and 220- loops^42^ that define specificity in the trypsin fold^43^ and re-organize in the Huber-Bode mechanism of zymogen activation^9^. The structures of I16T and D194A (Figs. 1 and 3) vouch for the importance of the I16-D194 ionic interaction in stabilizing the architecture of the 186- and 220- loops that define affinity and specificity for monovalent cation binding in thrombin^44,45^ and other trypsin-like proteases^46,47^. Perturbation of the Na^+^ binding site is particularly evident in the I16T mutant, where the ion pair between R187 and D222 is completely disrupted and the two side chains point in opposite directions. Consistent with this structural observation, the Na^+^ binding affinity of the I16T mutant is 70-fold lower than that of wild type (Table 2). A less extensive perturbation of the Na^+^ binding site is observed in the structure of the D194A mutant where a significant portion of the electron density of the 186-loop is missing, as in many zymogen structures^5,6^. The Na^+^ binding affinity of the D194A mutant is 5-fold lower than that of wild type and is significantly higher than that of the I16T mutant (Table 2).

**Table 2:** Hydrolysis of natural substrates and Na^+^ binding.

	Fibrinogen *k*_*cat*_/*K*_*m*_ (μM^−1^ s^−1^)	PAR1 *k*_*cat*_/*K*_*m*_ (μM^−1^ s^−1^)	Protein C^1^ *k*_*cat*_/*K*_*m*_ (mM^−1^ s^−1^)	Na^+^ *K*_*d*_ (mM)
wt	17 ± 1	32 ± 2	220 ± 10	16 ± 3
I16T	0.021 ± 0.001	0.034 ± 0.001	7.3 ± 0.1	1100 ± 300
D194A	0.0052 ± 0.0003	0.0059 ± 0.0002	0.15 ± 0.01	80 ± 20

^1^In the presence of 50 nM rabbit thrombomodulin. Experimental conditions described under Materials.

### Stability profiles

**Figure 5:**
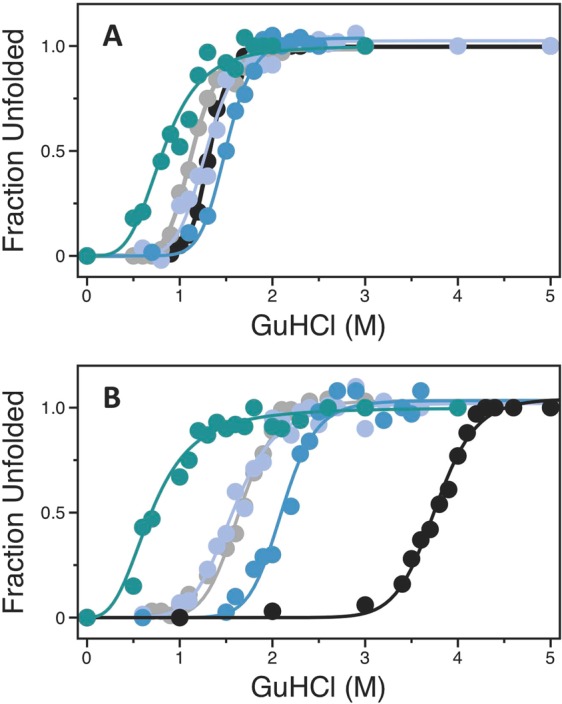
(**A,B**) Denaturation profiles of thrombin wild-type (black), mutants I16T (grey), D194A (purple) and D189A (blue), and zymogen prethrombin-2 (teal) measured in the absence (**A**) or presence (**B**) of the active site inhibitor PPACK. Midpoint values [GuHCl]_50_ measured in the absence of PPACK are: 1.32 ± 0.01 M (wild-type thrombin), 1.13 ± 0.01 M (I16T), 1.29 ± 0.02 M (D194A), 1.50 ± 0.01 M (D189A) and 0.85 ± 0.03 M (prethrombin-2). Midpoint values [GuHCl]_50_ measured in the presence of PPACK are: 3.75 ± 0.01 M (wild-type thrombin), 1.65 ± 0.02 M (I16T), 1.58 ± 0.02 M (D194A), 2.10 ± 0.02 M (D189A) and 0.73 ± 0.02 M (prethrombin-2). Experimental conditions are: 200 mM NaCl, 20 mM Tris, 10% glycerol, pH 7.5 at 20 °C.

nteresting observations emerge from investigation of the role of the I16-D194 interaction in the stability of the trypsin fold. The midpoints of GuHCl-induced denaturation curves for the two mutants I16T and D194A are comparable to that of wild-type, indicating that integrity of the I16-D194 ionic interaction does not contribute significantly to stability of the mature protease (Fig. 5A). The scenario changes in the presence of the irreversible inhibitor and transition state analog H-D-Phe-Pro-Arg-CH_2_Cl (PPACK) that makes extensive contacts with the active site^2,24^. Binding of PPACK is known to increase the stability of thrombin against chemical^48^ and thermal^49^ denaturation, but this effect is significantly reduced for the I16T and D194A mutants (Fig. 5B). The lack of PPACK-enhanced stability in these mutants is likely due to perturbation of the primary specificity pocket and is reproduced by the mutant D189A that selectively abrogates interaction with the Arg residue at the P1 position of the inhibitor^50^. A similar effect is observed with the immediate zymogen precursor of thrombin prethrombin-2, whose primary specificity pocket is incompletely structured^51^, and for which binding of PPACK fails to enhance stability against chemical denaturation. We conclude that an important contribution of the I16-D194 ionic interaction is to increase stability of the transition state by ordering the architecture of the primary specificity pocket. No such effect is produced in the free form of the enzyme.

### Activation of physiological substrates

The specificity constants of the I16T and D194A variants for the hydrolysis of natural substrates measured under physiological conditions are reported in Table 2. The D194A mutant cleaves procoagulant (fibrinogen), prothrombotic (PAR1) and anticoagulant (protein C) substrates with *k*_cat_/*K*_m_ values that are several thousand times slower than those of wild-type. The loss of activity is likely due to a significant perturbation of the primary specificity pocket as documented by the crystal structure (Figs. 1–4) and stability measurements (Fig. 5). The I16T mutant also features a significant drop in activity toward fibrinogen and PAR1, with *k*_cat_/*K*_m_ values about 1000-fold lower than those of wild-type (Table 2). On the other hand, cleavage of protein C in the presence of thrombomodulin is reduced to less extent (30-fold) because the cofactor corrects some of the structural defects produced by the mutation. Notably, the analogous I16L mutant of clotting factor Xa is a very poor enzyme, but its catalytic activity is almost completely restored when assembled with the cofactor Va on platelet membranes^20,22^. However, it is unclear why thrombomodulin fails to rescue the defects generated upon the D194A substitution.

### Mechanism of binding

**Figure 6:**
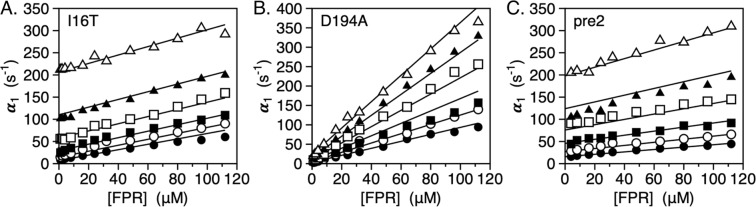
(**A–C**) Rapid kinetics of FPR binding to the thrombin mutants I16, D194A and the zymogen precursor prethrombin-2, all carrying the S195A substitution, over the temperature range 5–30 °C. Continuous lines were drawn according to Eq. 4 in the text with each rate constant expressed as in Eq. 5 and represent a global fit of the entire data set with best-fit parameter values listed in Table 3. The values of the rate constants are at the reference temperature *T*_0_ = 288.15 K (15 °C). Experimental conditions are: 400 mM ChCl, 50 mM Tris, 0.1% PEG8000, pH 8.0 at 5 °C (closed circles), 10 °C (open circles), 15 °C (closed squares), 20 °C (open squares), 25 °C (closed triangles), 30 °C (open triangles).

Binding of ligand to the active site of protease and zymogen obeys the mechanism of conformational selection (Eq. 1) where a pre-existing equilibrium between E* and E precedes the binding interaction^7,8^. Recent measurements of the binding of the tripeptide substrate H-D-Phe-Pro-Arg-p-nitroanilide (FPR) to the S195A mutant of thrombin and its zymogen precursors by rapid kinetics have quantified the E*-E distribution along the activation pathway^8^. Specifically, the transition from prothrombin to thrombin increases the population of the E form and drastically reduces the value of *k*_*off*_, thereby producing an environment that promotes substrate binding and catalysis. Figure 6 shows the results of analogous rapid kinetics measurements carried out with the I16T and D194A mutants, carrying the additional substitution S195A in the active site to prevent hydrolysis of FPR. The results are compared to those of the immediate zymogen precursor of thrombin, prethrombin-2, where the R15-I16 peptide bond remains intact and the I16-D194 ionic interaction cannot form. Mutation of I16 and D194 profoundly affects FPR binding and produces a simple lock-and-key mechanism of recognition (Eq. 3 and Fig. 6). The E*-E equilibrium detected in the wild-type is no longer in place when the I16-D194 ionic interaction is compromised. The conformation of the enzyme freezes in an intermediate state that binds ligand at the active site as a rigid body association. Other features of the two mutants are revealed by the temperature dependence of the relaxations that remain linear and without indication of conformational transitions preceding and/or following binding. The mutant I16T resembles the properties of prethrombin-2 the most, with similar values of *k*_*on*_ and *k*_*off*_ at the reference temperature of 15 °C (Table 3) but different activation energies. The resulting equilibrium dissociation constant for the mutant is 40 ± 4 μM compared to 100 ± 10 μM in prethrombin-2 and the associated enthalpy of binding is −19 ± 1 kcal/mol compared to −8.4 ± 0.7 kcal/mol. In contrast, the mutant D194A features significantly different rate constants at the reference temperature, especially *k*_*off*_, that translate in an equilibrium dissociation constant for FPR binding of 4.9 ± 0.5 μM. The value is 20-fold higher than that of wild-type measured recently under identical solution conditions^8^, but is significantly lower than that of the I16T mutant or prethrombin-2 (Table 3). The higher affinity of the D194A mutant compared to I16T may be explained by the lack of disruption of the architecture of the oxyanion hole. It also proves that lack of the I16-D194 ionic interaction in the mature enzyme compromises function but does not necessarily produce an environment that is structurally and energetically equivalent to the zymogen prethrombin-2. This conclusion is further supported by the fact that perturbation of the I16-D194 H-bond has energetic consequences that depend on how the ionic interaction is disrupted. The enthalpy of FPR binding to the D194A mutant is −4.1 ± 0.5 kcal/mol, or 15 kcal/mol less exothermic than that measured for prethrombin-2 and 4 kcal/mol less exothermic compared to the I16T mutant. Given the differences in binding affinity between the mutants, it is important to note that FPR binding to I16T takes place with an entropy cost (−11 ± 1 cal/mol/K) that is considerably smaller than that of prethrombin-2 (−39 ± 3 cal/mol/K) but FPR binding to the D194A mutant is entropy-driven (+10 ± 1 cal/mol/K).

**Table 3:** Kinetic rate constants, activation energies and thermodynamic parameters for FPR binding.

	*k*_*off*_ (s^−1^)	*k*_*on*_ (μM^−1^ s^−1^)	*E*_*off*_ (kcal/mol)	*E*_*on*_ (kcal/mol)	*k*_*d*_ (μM)	Δ*G* (kcal/mol)	Δ*H* (kcal/mol)	Δ*S* (cal/mol/K)
Pre2	46 ± 4	0.46 ± 0.05	17 ± 1	8.6 ± 0.5	100 ± 10	−5.3 ± 0.2	−8.4 ± 0.5	−11 ± 1
I16T	29 ± 3	0.73 ± 0.07	23 ± 1	3.0 ± 0.2	40 ± 4	−5.8 ± 0.2	−17 ± 1	−39 ± 3
D194A	7.8 ± 0.6	1.6 ± 0.1	13 ± 1	8.9 ± 0.4	4.9 ± 0.5	−7.0 ± 0.3	−4.1 ± 0.2	+10 ± 1
wt	0.6 ± 0.1	2.6 ± 0.3	—	—	0.23 ± 0.02	−8.8 ± 0.3	—	—

Experimental conditions are: 400 mM ChCl, 50 mM Tris, 0.1% PEG8000, pH 8.0. Values of rate constants are at the reference temperature *T*_0_ = 288.15 K (15 °C). Definitions are: $\,{K}_{d}=\frac{{k}_{off}}{{k}_{on}}$ (equilibrium dissociation constant); Δ*G* = *RTlnK*_*d*_ (binding free energy); Δ*H* = *E*_*on*_ − *E*_*off*_ (binding enthalpy); $\Delta S=\frac{\Delta H-\Delta G}{T}$ (binding entropy). Values for wild-type are from previously published work^8^.

## Discussion

Activity in trypsin-like proteases ensues via a mechanism first proposed by Huber and Bode^9^ that involves a proteolytic cut at R15 to generate a new N-terminus that interacts with the highly conserved D194 next to the catalytic S195 and organizes both the oxyanion hole and primary specificity pocket. This widely accepted mechanism pictures the zymogen as an inactive precursor of the mature protease because of its incorrect architecture to promote binding and catalysis^3,9^. However, not all members of the trypsin family conform to this paradigm. The trypsin fold has features not captured by the Huber-Bode mechanism of activation, most notably a pre-existing allosteric equilibrium between two conformations, E* and E, that differ in accessibility of the active site region^5,6^. The E*-E equilibrium layers on top of the Huber-Bode mechanism and reveals that the transition from zymogen to protease is linked to redistribution of the population of E* and E forms. Typically, the zymogen exists predominantly in the E* form where access to the primary specificity pocket is hindered by collapse of the 215–217 segment into the active site. Activation progressively shifts the equilibrium from E* to E, opens access to the active site by repositioning the 215–217 segment and reduces the rate of ligand dissociation^8^, leading to efficient binding and catalysis. We therefore addressed the structural and functional consequences of perturbing the I16-D194 interaction to test widely accepted assumptions and any linkage between the Huber-Bode mechanism and the E*-E equilibrium.

Residue I16 was replaced with Thr because a polar side chain of size similar to Ile is not documented in any existing trypsin-like protease. Residue D194 was perturbed more drastically by replacing the negatively charged side chain with the Ala substitution. The X-ray crystal structure of the I16T mutant reveals a rearrangement of D194 toward the T16 polar side chain, with a resulting flip of the 192–193 peptide bond and disruption of the oxyanion hole. The flip is caused by a slight shift of residue E192 that breaks the N143-E192 backbone H-bond as observed in the E* form of thrombin^25,29^, complement factor B^33^, the arterivirus nsp4^32^, the epidermolyic toxin A^31^ and clotting factor VIIa^35^ The side chain of E192, an uncompensated negative charge guarding access to the active site, assumes a conformation that contributes to occlusion of the primary specificity pocket by pulling on the entire 215–217 segment as observed in other thrombin mutants with compromised activity^27,30^. The crystal structure of D194A offers an additional view of the consequences of perturbing the critical I16-D194 ionic interaction. Unexpectedly, the conformation of the 192–193 peptide bond and the structural integrity of the oxyanion hole in the D194A mutant are preserved even though the highly conserved H-bond between N143 and E192 is perturbed. The backbone N atom of G193 remains correctly aligned with that of S195 for optimal binding of substrate in the transition state because of a compensatory H-bond established with G43. This observation refines the role for the I16-D194 ionic interaction of the Huber-Bode mechanism in structuring the oxyanion hole. Most likely, the linkage is indirect and mediated by other molecular features that are disrupted differently with the I16T and D194A mutations.

Additional support to the different perturbation of structure caused by mutation of I16 and D194 comes from rapid kinetics measurements of FPR binding to the active site. In both cases, the E*-E equilibrium is abrogated and binding obeys a simple lock-and-key mechanism of recognition over a wide temperature range (5–30 °C). The same result is obtained with the immediate zymogen precursor of thrombin, prethrombin-2. Importantly, the mutant I16T is functionally more similar to prethrombin-2 than D194A. The FPR binding affinity of the D194A mutant is 20-fold higher than that of I16T or prethrombin-2 and the enthalpy of binding is significantly less exothermic. We conclude that the I16-D194 ionic interaction is necessary to establish the E*-E equilibrium in the mature protease. Without this interaction, the conformation locks into an intermediate state that binds ligand at the active site according to a rigid body association.

A final point of interest is that the I16-D194 ionic interaction contributes little to stability of the fold of the mature protease in the free form. The major contribution of the H-bond is to stabilize the transition state and the environment of the primary specificity pocket around D189. The intermediate “rigid” conformation generated upon abrogation of the I16-D194 H-bond is as stable as the E* and E forms, but fails to gain stability upon binding of PPACK to the active site because it lacks the full complementarity of the E form when bound to the ligand.

## Methods

### Reagents

A Quick-Change Lightning site-directed mutagenesis kit was used to introduce the mutations of interest according to a standard protocol supplied by the manufacturer (Agilent Technology). The I16T and D189A variants were initially purified as prethrombin-2^52^, while D194A was purified as prethrombin 1^53^. Zymogens were converted to thrombin by overnight incubation at ambient temperature with 0.5 units Ecarin (Sigma Aldrich) per mg protein. The activation process was confirmed by SDS-PAGE analyses and the activated protein was purified on a Heparin-sepharose column (GE Healthcare) equilibrated with 100 mM NaCl, 20 mM Tris, pH 7.5 and eluted with a 0–1 M NaCl gradient. The irreversible inhibitor H-D-Phe-Pro-Arg-CH_2_Cl (PPACK) was purchased from Haematological Technologies. PPACK is a relevant probe of the active site as documented by detailed structural information^2,24^.

### X-ray studies

The thrombin mutants I16T and D194A were crystallized at 20 °C by the vapor diffusion technique, using an Art Robbins Instruments Phoenix liquid handling robot and mixing equal volume of protein (10 mg/ml) and reservoir solution. Optimization of crystal growth was achieved by the hanging drop vapor diffusion method. Diffraction quality crystals were grown at 20 °C and then frozen in 25% glycerol using the original mother liquid. Data for these two structures were collected at 100° K with a home source (Rigaku 1.2 kw MMX007 generator with VHF optics) Rigaku Raxis IV++ detector and were indexed, integrated and scaled with the HKL2000 software package^54^. The structures were solved by molecular replacement using PHASER from the CCP4 suite^55^ and the structure of thrombin complexed with PPACK (PDB ID: 1PPB)^2^ as a starting model. Refinement and electron density generation were done using REFMAC5 from the CCP4 package. 5% of the reflections were randomly selected as a test set for cross-validation for these two structures. Model building and analysis of the two structures were carried out using COOT^56^. Ramachandran plots were calculated using PROCHECK^57^. Statistics for data collection and refinement are summarized in Table 1. Atomic coordinates and structure factors have been deposited in Protein Data Bank (PDB ID: 6PXJ for I16T and 6PXQ for D194A).

### Functional studies

Values of *k*_cat_/*K*_m_ for the cleavage of fibrinogen and a PAR1 fragment and activation of protein C in the presence of 50 nM rabbit thrombomodulin (Haematologic Technologies) were determined as reported^52^ under experimental conditions: 145 mM NaCl, 0.1% PEG 8000, 20 mM Tris, pH 7.5 at 37 °C.

Binding of Na^+^ was performed by equilibrium titration of intrinsic fluorescence as reported^44^ under experimental conditions: 800 mM ChCl, 0.1% PEG 8000, 20 mM Tris, pH 8 at 25 °C. All kinetic and equilibrium measurements were done at least in duplicate and standard errors were below 5%.

Equilibrium denaturation measurements were carried out by incubating 200 nM of protein with various concentrations of GuHCl (Ultra-pure grade, Invitrogen) for 30 min at 20 °C. For measurements carried out in the presence of inhibitor, the protein (10 μM) was incubated with 40 to 50-fold molar excess of PPACK (HaemTech) at 20 °C until no activity against cleavage of chromogenic substrate was noticeable. Typically, the thrombin variants were completely saturated with inhibitor after incubation up to 4 h. The zymogen prethrombin-2 also binds PPACK covalently^58^ and was incubated with 250-fold molar excess of the inhibitor for 24 hr. The inhibited proteins were then diluted to a final concentration of 200 nM and equilibrated with various concentrations of GuHCl at 20 °C for 30 min. Equilibrium of unfolding was established during incubation. The GuHCl-induced denaturation was monitored at 20 °C by following changes in fluorescence emission at 336 nm after exciting at 280 nm in a cell with a path length of 0.3 cm. The denaturation midpoint was obtained from fitting the fraction of unfolded molecules as described^59^. All experiments were done at least in duplicate under experimental conditions: 200 mM NaCl, 10% glycerol, 20 mM Tris, pH 7.5 at 20 °C.

### Rapid kinetics of binding

A detailed discussion of binding mechanisms studied by rapid kinetics is given elsewhere^60–62^, and is summarized below for the case of trypsin-like proteases. Ligand binding to the active site of a trypsin-like protease or zymogen is the conformational selection mechanism^7,61^

1 $${E}^{\ast }\begin{array}{c}{k}_{12}\\ \rightleftarrows \\ {k}_{21}\end{array}E\begin{array}{c}{k}_{on}[{\rm{L}}]\\ \rightleftarrows \\ {k}_{off}\end{array}E:L$$

E* and E depict the pre-existing conformations with active site accessible (E) or inaccessible (E*) to ligand binding that interconvert with first-order rate constants *k*_12_ and *k*_21_. The ratio *k*_21_/*k*_12_ gives the E*:E partitioning between the two forms. The ligand, L, selectively binds to E with a second-order rate of association *k*_*on*_ and dissociates with a first-order rate *k*_*off*_. Under conditions where L is in large excess over the macromolecule, the reaction scheme in Eq. 1 gives two independent rates of relaxation to equilibrium according to the expression

2 $$2{\alpha }_{1,2}={k}_{12}+{k}_{21}+{k}_{on}[{\rm{L}}]+{k}_{off}\pm \sqrt{{({k}_{on}[{\rm{L}}]+{k}_{off}-{k}_{12}-{k}_{21})}^{2}+4{k}_{21}{k}_{on}[{\rm{L}}]}$$

The fast relaxation, *α*_1_ (+sign in Eq. 2), reflects the binding event and eventually grows linearly with [L]. The slow relaxation, *α*_2_ (−sign in Eq. 2) always saturates for high [L] and reflects the conformational transition associated with binding.

When ligand binding does not involve conformational transitions, as in a rigid body association, recognition takes place according to the lock-and-key mechanism

3 $$E\begin{array}{c}{k}_{on}[{\rm{L}}]\\ \rightleftarrows \\ {k}_{off}\end{array}E:L$$

A single relaxation is obtained in this case because letting *k*_12_ and *k*_21_ equal to zero in Eq. 2 gives

4 $${\alpha }_{1}={k}_{off}+{k}_{on}[{\rm{L}}]$$

Measurements carried out as a function of temperature, from 5 to 30 °C, were analyzed globally by expressing each rate constant in Eq. 4 according to the Arrhenius equation

5 $$k={k}_{0}\,\exp \{-\frac{E}{R}(\frac{1}{T}-\frac{1}{{T}_{0}})\}$$

Where *k*_0_ is the value of *k* at the reference temperature *T*_0_, *E* is the activation energy and *R* the gas constant.

Rapid kinetic experiments of FPR binding were conducted on an Applied Photophysics SX20 stopped-flow spectrometer using an excitation of 295 nm and a cutoff filter at 320 nm over the temperature range 5–30 °C. The dead time of the mixing cell for this instrument is 0.5–1 ms. Final concentrations of 150–250 nM prethrombin-2 or thrombin mutants I16T and D194A, all carrying the S195A substitution, were used in a buffer containing 50 mM Tris, 0.1% PEG8000, 400 mM ChCl, pH 8.0 at the desired temperature. The solution containing the protein was mixed 1:1 with 60 µL solutions of FPR in the same buffer. Baselines were measured by mixing the protein into buffer in the absence of ligand. Each kinetic trace was taken as the average of at least ten determinations and fit to single or double exponentials based on the analysis of residuals using software supplied by Applied Photophysics. Values of the relaxations as a function of [FPR] and temperature were fit globally to extract the values of kinetic rate constants (Eq. 4) and activation energies (Eq. 5).

## Data Availability

Recombinant reagents and data presented in this study are available from the corresponding author upon reasonable request.
